# Morphology and molecular study of three new Cordycipitoid fungi and its related species collected from Jilin Province, northeast China

**DOI:** 10.3897/mycokeys.83.72325

**Published:** 2021-09-27

**Authors:** Jia-Jun Hu, Gui-Ping Zhao, Yong-Lan Tuo, Dan Dai, Di-Zhe Guo, Gu Rao, Zheng-Xiang Qi, Zhen-Hao Zhang, Yu Li, Bo Zhang

**Affiliations:** 1 School of Life Science, Northeast Normal University, Changchun City, 130024, Jilin Province, China Jilin Agricultural University Changchun China; 2 Engineering Research Centre of Edible and Medicinal Fungi, Ministry of Education, Jilin Agricultural University, Changchun City, 130118, Jilin Province, China Northeast Normal University Changchun China; 3 Hebei Normal University of Science and Technology, Qinghuangdao City, 066004, Hebei Province, China Hebei Normal University of Science and Technology Qinghuangdao China

**Keywords:** Cordyceps, host, new species, phylogenetic study, relationship

## Abstract

*Cordyceps* species are notable medicinal fungi in China, which are pathogenic on insects and exhibit high biodiversity in tropical and subtropical regions. Recently, three new *Cordyceps* specie*s*, *Cordycepschangchunensis* and *Cordycepsjingyuetanensis* growing on pupae of Lepidoptera and *Cordycepschangbaiensis* growing on larvae of Lepidoptera, were found in Jilin Province, China and are described, based on morphological and ecological characteristics. These three new species are similar to the *Cordycepsmilitaris* group, but are distinctly distinguishable from the known species. *Cordycepschangchunensis*, characterised by its small and light yellow to orange stromata which is occasionally forked, covered with white mycelium at the base of stipe, globose to ovoid perithecia, is macroscopically similar to *Cordycepsmilitaris*. *Cordycepschangbaiensis* is clearly discriminated from other *Cordyceps* species by its white to orange and branched stromata, clavate to cylindrical fertile apical portion, immersed and globose to ovoid perithecia. Moreover, unbranched, clavate and orange to light red stromata, almond-shaped to ovoid and immersed perithecia separate *Cordycepsjingyuetanensis* from other *Cordyceps* species. nrITS, nrLSU and EF-1α sequences were undertaken and phylogenetic trees, based on Maximum Likelihood and Bayesian Inference analysis showed that the three new species clustered with *Cordycepsmilitaris*, but formed individual clades, as well as confirmed the results of our morphological study.

## Introduction

The family Cordycipitaceae belongs to Hypocreales with plant-, animal- and fungus-based nutrition modes (Sung et al. 2007; Vega et al. 2009). The species of Cordycipitaceae are a wide variety which infect invertebrates and, in the tropics and subtropics, are known to have the highest species diversity (Kobayasi 1941, 1982). According to current data, over 900 species of Cordycipitoid fungi are reported worldwide (Yan and Bau 2015; Zha et al. 2018). In China, more than 146 species are recorded (Yan and Bau 2015).

Cordycipitoid fungi were first described in 1753 as *Clavariamilitaris* L., later being recognised as *Cordycepsmilitaris* (L.) Fr. The genus *Cordyceps* Fr. was established by Fries in 1818, encompassing over 450 species (Kobayasi 1982; Luangsa-ard et al. 2007). Compared with a large number of species, subdivisions into infrageneric groups, for example, subgenera and sections, have been proposed in the *Cordyceps* classification, traditionally based on morphological and ecological characters (Stensrud et al. 2005). The classification of *Cordyceps*, based on the studies of Kobayasi (1941, 1983), three subgenera, C.subg.Cordyceps, C.subg.Ophiocordyceps and C.subg.Neocordyceps were recognised. Subg. Cordyceps was characterised by the production of either immersed or superficial perithecia, which are approximately at right angles to the surface of stroma and ascospores break into part-spores at maturity. Mains proposed a different viewpoint, two subgenera, C.subg.Cryptocordyceps and C.subg.Racemella, were added (Mains 1958). Based on nrITS, nrSSU, nrLSU, EF-1α, RPB1, RPB2, TUB and ATP6 sequences, the phylogenetic study implied that the Cordycipitoid fungi belong to six genera (*Cordyceps* Fr., *Metacordyceps* G.H. Sung, J.M. Sung, Hywel-Jones & Spatafora, *Tyrannicordyceps* Kepler & Spatafora, *Elaphocordyceps* G.H. Sung & Spatafora, *Ophiocordyceps* Petch and *Polycephalomyces* Kobayasi) across three families, Cordycipitaceae, Clavicipitaceae and Ophiocordycipitaceae (Sung et al. 2007; Yan and Bau 2015).

The host of Cordycipitoid fungi is varied and the fungi are always parasitic on larvae of swifts, pupae of Lepidoptera, spiders etc. Cordycipitoid fungi have a strong relationship with the environment and its host (Zha et al. 2019).

In this study, three new species of *Cordyceps* are reported, based on morphology and molecular studies. Furthermore, the relationship between the host and *Cordyceps* species is analysed.

## Material and methods

### Sampling and morphological studies

The specimens were photographed in situ. The size of the stromata was measured when fresh. After examination and description of the fresh macroscopic characters, the specimens were dried in an electric drier at 40–45 °C.

Descriptions of macroscopic characters were based on field notes and photographs. The colours correspond to the “Flora of British fungi: colour identification chart” (Royal Botanic Garden 1969). The dried specimens were rehydrated in 94% ethanol for microscopic examination and then mounted in 3% potassium hydroxide (KOH), 1% Congo Red, Cotton Blue and Melzer’s Reagent (Torres et al. 2005), along with a Zeiss Axio Lab. A1 microscope for observation. For each species, a minimum of 40 part-spores was measured from two different ascocarps, part-spores are given as length × width (l × w). The specimens examined are deposited in the Herbarium of Mycology of Jilin Agricultural University (HMJAU).

### DNA extraction, PCR amplification and sequencing

Total DNA was extracted from dried specimens using the NuClean Plant Genomic DNA Kit (Kangwei Century Biotechnology Company Limited, Beijing, China). Sequences of the internal transcribed spacer region (ITS), nuclear large ribosomal subunits (LSU) and translation elongation factor 1-alpha (EF-1α) were used for phylogenetic analysis. The ITS sequence was ampliﬁed using the primer pair ITS4 and ITS5 (White et al. 1990), LSU sequence was ampliﬁed using the primer pair LROR and LR7 (Stensrud et al. 2005) and EF-1α sequence was ampliﬁed using the primer pair 983F and 2218R (Castlebury et al. 2004).

Reaction programmes followed Yan and Bau (2015), Castillo et al. (2018) and Ban et al. (2015), respectively. PCR products were visualised via UV light after electrophoresis on 1% agarose gels stained with ethidium bromide and purified using Genview High-Efficiency Agarose Gels DNA Purification Kit (Gen-View Scientific Inc., Galveston, TX, USA). The purified PCR products were sent to Sangon Biotech Limited Company (Shanghai, China) for sequencing using the Sanger method. The new sequences were deposited in GenBank.

### Data analysis

Based on the results of BLAST and morphological similarities, the sequences obtained and related to these samples are listed in Table 1. A dataset comprising of sequences from this study, 31 representative sequences showing the highest similarity to *Cordyceps* spp. and the outgroup *Metacordycepstaii* (Z.Q. Liang & A.Y. Liu) G.H. Sung, J.M. Sung, Hywel-Jones & Spatafora, *Metarhiziumyongmunense* (G.H. Sung, J.M. Sung & Spatafora) Kepler, S.A. Rehner & Humber, *Nigeliamartiale* (Speg.) Luangsa-ard & Thanakitp., *Ophiocordyceps* spp. and *Tolypocladiumophioglossoides* (J.F. Gmel.) C.A. Quandt, Kepler & Spatafora, retrieved from GenBank, were aligned with using ClustalX (Thompson et al. 1997), MACSE V2.03 (Ranwez et al. 2018) and MAFFT (Katoh and Standley 2013), then manually adjusted in BioEdit (Hall 1999). The datasets were aligned first and then, nrITS, nrLSU and EF-1α sequences were combined with Mesquite. The tree construction procedure was performed in PAUP* version 4.0b10 (Swofford 2002) as described by Jiang et al. (Jiang et al. 2011). All characters were equally weighted and gaps were treated as missing data.

**Table 1.** Voucher information and GenBank accession numbers of ITS, LSU and EF-1α DNA sequences of *Cordycepschangchunensis*, *Cordycepschangbaiensis*, *Cordycepsjingyuetanensis* and related species used in this study.

Species name	Specimen/Strain number	Host/Substratum	GenBank accession numbers			References
			ITS	LSU	EF-1α	
* Akanthomyces lecanii *	CBS101247	Homopteran	JN049836	AF339555	DQ522359	(Kepler et al. 2012)
* A. tuberculatus *	NBRC106949	Lepidoptera	JN943318	JN941400	MF416490	(Kepler et al. 2017; Schoch et al. 2012)
* Blackwellomyces cardinalis *	CBS113414	Lepidoptera	MH862930	MH874497	EF469059	(Sung et al. 2007; Vu et al. 2019)
* B. pseudomilitaris *	NBRC101411	Lepidoptera	JN943308	JN941395	MT017849	(Mongkolsamrit et al. 2020; Schoch et al. 2012)
* Cordyceps bassiana *	IFO4848	Lepidoptera	AB027382	AB027382	MN401498	(Khonsanit et al. 2020; Nikoh and Fukatsu 2000)
* C. bifusispora *	ARS5690/EFCC8260	Lepidoptera	AY245627	EF468807	EF468747	(Kuo et al. 2005; Sung et al. 2007)
* C. brongniartii *	NBRC101395	Lepidopteran pupae	JN943298	JN941382	JF416009	(Kepler et al. 2012; Schoch et al. 2012)
* C. cateniobliqua *	CBS153.83	Lepidoptera	MH861560		MT017860	(Vu et al. 2019)
*** C. changbaiensis ***	**HMJAU48255**	** Lepidoptera **	**MW893252**	**MW893277**	**MZ616772**	**This study**
*** C. changbaiensis ***	**HMJAU48260**	** Lepidoptera **	**MW893270**	**MW893272**	**MZ616774**	**This study**
*** C. changchunensis ***	**HMJAU48251**	** Lepidoptera **	**MW893249**	**MW893274**	**MZ616769**	**This study**
*** C. changchunensis ***	**HMJAU48252**	** Lepidoptera **	**MW893250**	**MW893275**	**MZ616775**	**This study**
*** C. changchunensis ***	**HMJAU48259**	** Lepidoptera **	**MW893251**	**MW893276**	**MZ616773**	**This study**
* C. chiangdaoensis *	BCC75734/TBRC7274	Coleopteran larvae	KT261394	MF140732	KT261404	(Mongkolsamrit et al. 2018; Tasanathai et al. 2016)
* C. coleopterorum *	CBS110.73	Coleoptera	AY624177	JF415988	JF416028	(Kepler et al. 2012; Luangsa-Ard et al. 2005)
* C. exasperata *	MCA2155	Lepidoptera		MF416542	MF416486	(Kepler et al. 2017)
* C. farinosa *	CBS111113	Lepidoptera	AY624181	MF416554	MF416499	(Kepler et al. 2017; Luangsa-Ard et al. 2005)
* C. fumosorosea *	CBS244.31	Coleoptera	AY624182	MF416557	MF416503	(Kepler et al. 2017; Luangsa-Ard et al. 2005)
* C. hepialidicola *		Lepidoptera	AF315649			Unpublished
*** C. jingyuetanensis ***	**HMJAU48253**	** Lepidoptera **	**MW893253**	**MW893278**	**MZ616770**	**This study**
*** C. jingyuetanensis ***	**HMJAU48261**	** Lepidoptera **	**MW893271**	**MW893273**		**This study**
* C. kyushuensis *	HMAS78115	Lepidoptera	EF368021	EF468813	EF468754	(Sung et al. 2007; Wang et al. 2008)
* C. militaris *	OSC93623	Lepidopteran pupae	JN049825	AY184966	DQ522332	(Sung et al. 2007)
*** C. militaris ***	**HMJAU48256**	**Lepidopteran pupae**	**MW888227**	**MW893279**		**This study**
* C. morakotii *	BCC55820/TBRC7276	Hymenoptera	KT261389	MF140731	KT261399	(Mongkolsamrit et al. 2018; Tasanathai et al. 2016)
* C. ninchukispora *	BCC30937	Lepidoptera	FJ765274	FJ765242	MF416477	(Kepler et al. 2017)
* C. ningxiaensis *	HMJAU25074	Diptera	KF309668	KF309671		(Yan and Bau 2015)
* C. polyarthra *	6578	Lepidoptera	AJ536548			Unpublished
* C. pruinosa *	ARSEF5413	Lepidoptera	JN049826	MK761215	DQ522351	(Kepler et al. 2012; Zha et al. 2019)
* C. qingchengensis *	MFLU17-1022	Lepidoptera	KY423506	MK761211	MK770630	(Zha et al. 2019)
* C. rosea *	Spat09-053	Lepidoptera		MF416536	MF416480	(Kepler et al. 2017)
* C. roseostromata *	ARSEF4870	Larva, not specified	AY245637	AF339523		(Kuo et al. 2005; Sung et al. 2001)
* C. scarabaeicola *	ARSEF5689	Coleoptera	JN049827	AF339524	DQ522335	(Kepler et al. 2012; Sung et al. 2007)
* C. scarabaeicola *	Arsef5689	Coleoptera	JN049827	AF339524		(Kepler et al. 2012; Sung et al. 2001)
***Cordyceps*** sp.	**HMJAU48254**	** Lepidoptera **	**MW888228**	**MW893280**	**MZ616771**	**This study**
* C. spegazzinii *	ARSEF7850	Diptera	DQ196435	DQ196435	GU734752	(Torres et al. 2005)
* C. taishanensis *	A-1	Lepidoptera	FJ008927			Unpublished
* C. tenuipes *	TBRC7266	Lepidoptera	MF140742		MF140828	(Mongkolsamrit et al. 2018; Vu et al. 2019)
* Isaria cicadae *	GACP07071701	Hemiptera	KX017277	MK761212	MT268245	(Zhi et al. 2021)
* I. japonica *	BCC2808	Lepidoptera	AY624199			(Luangsa-Ard et al. 2005)
* Metarhizium yongmunense *	EFCC2131	Lepidoptera	JN049856	EF468833	EF468770	(Kepler et al. 2012; Sung et al. 2007)
* Metacordyceps taii *	ARSEF5714	Lepidoptera	JN049829	AF543787	AF543775	(Sung et al. 2007)
* Nigelia martiale *	HMAS197472(S)	Coleoptera	JN049881	JF415975	JF416016	(Kepler et al. 2012)
* Ophiocordyceps acicularis *	OSC12858/OSC110987	Coleoptera	JN049820	DQ518757	DQ522326	(Kepler et al. 2012)
* O. clavata *	NBRC106961	Coleoptera	JN943327	JN941414	MH879672	(Schoch et al. 2012)
* O. gracilis *	EFCC8572	Lepidoptera	HM142942	EF468811	EF468751	(Sung et al. 2007; Zhong et al. 2010)
* O. rubiginosoperitheciata *	NBRC106966	Coleoptera	JN943344	JN941437		(Schoch et al. 2012)
* O. sinensis *	ARSEF6282	Lepidopteran pupae	HM595981	HM595885	EF468767	(Chan et al. 2011; Sung et al. 2007)
* Tolypocladium ophioglossoides *	NBRC106331	*Elaphomyces* sp.	JN943320	JN941408		(Schoch et al. 2012)

MrModeltest 2.3 was used to determine the best fitting substitution model for each dataset for Bayesian Inference, which was calculated with MrBayes 3.2.6 with a general time-reversible DNA substitution model and a gamma distribution rate variation across sites (Ronquist and Huelsenbeck 2003). Four Markov chains were run for two runs from random starting trees for four million generations until the split deviation frequency value was < 0.01 and trees were sampled every 100 generations. raxmlGUI 2.0 (Edler et al. 2020) was used for Maximum Likelihood (ML) analysis with 1,000 bootstrap replicates using the GTRGAMMA algorithm to perform a tree inference and search for optimal topology (Vizzini et al. 2015).

## Results

### Phylogenetic analysis

**Figure 1.**
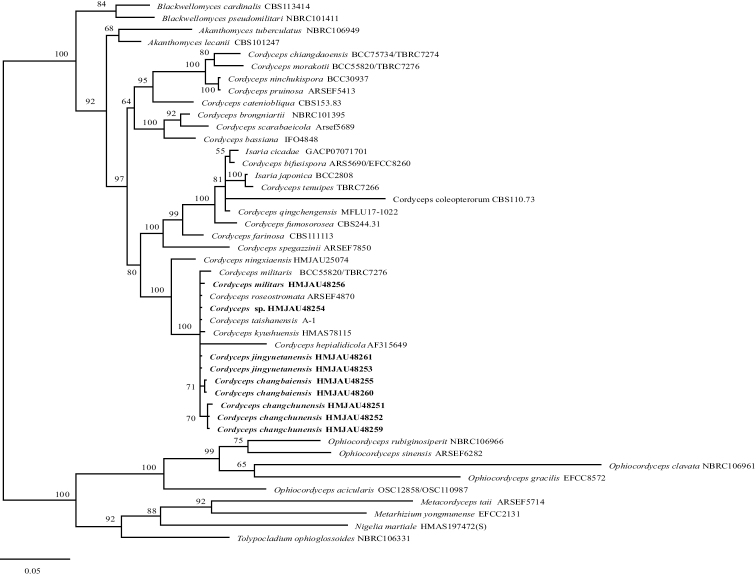
Phylogenetic tree of Cordycepitiod fungi, based on ITS from Bayesian analysis; self-examined sequences are shown in bold.

The phylogenetic tree, based on ITS from Bayesian analysis, included sequences from 46 fungal samples representing 43 taxa and the results are shown in Fig. 1. According to the phylogenetic tree, the three new species gather into one branch with *C.militaris*, *C.roseostromata* Kobayasi & Shimizu, *C.taishanensis* B. Liu, P.G. Yuan & J.Z. Cao, *C.kyushuensis* A. Kawam. and *C.hepialidicola* Kobayasi & Shimizu, but the species *C.jingyuetanensis* does not gather into one branch by itself. Meanwhile, the genus *Cordyceps* was divided into three independent clades. Furthermore, *Cordyceps* and *Akanthomyces* Lebert are a sister clade to *Blackwellomyces* Spatafora & Luangsa-ard.

**Figure 2.**
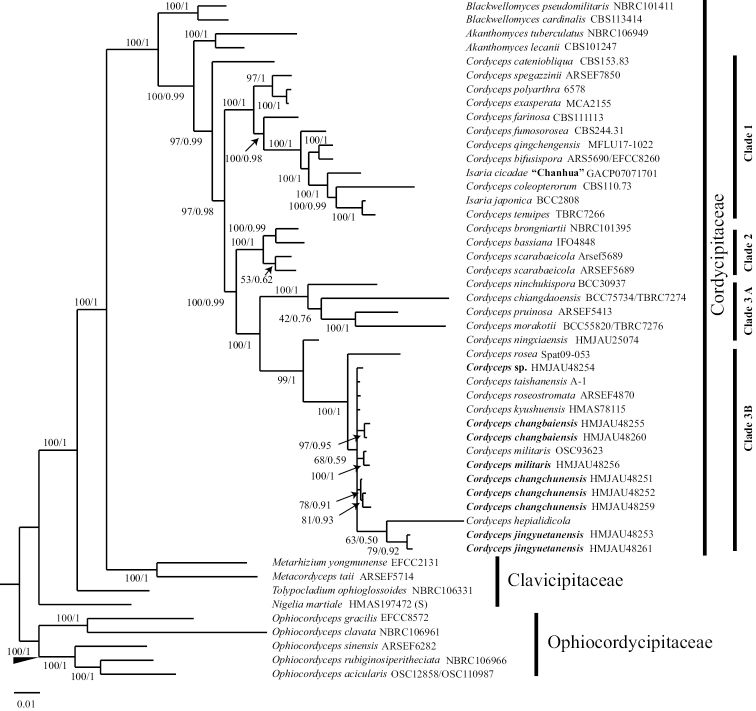
Phylogenetic tree of Cordycepitiod fungi, based on concatenated ITS, LSU and EF-1α from Bayesian analysis and Maximum Likelihood analysis; self-examined sequences are shown in bold.

For these reasons, the combined ITS, LSU and EF-1α dataset including 121 fungal samples representing 48 taxa was used for analysis and the results are shown in Fig. 2. In these data, the three new species are in three independent clades included in the *C.militaris* complex, *C.jingyuetanensis* was close to *C.hepialidicola* Kobayasi & Shimizu and is different from Fig. 1. From the phylogenetic tree (Fig. 2), the species of *Cordyceps* are mainly divided into three independent clades. Moreover, the family Cordycipitaceae clustered into three clades and the genus *Akanthomyces* formed a sister clade to the genus *Cordyceps*.

### Taxonomy

#### Cordyceps changchunensis

Taxon classificationFungiHypocrealesCordycipitaceae

J.J. Hu, Bo Zhang & Y. Li sp. nov.

18BCF603-5E06-5BAC-9970-8F1423685B46

839249

Figs 3 , 4

##### Holotype.

China. Jilin Province: Changchun City, Jingyuetan National Forest Park, 43.77°N, 125.47°E, 27 August 2018, Jia-Jun Hu, Bo Zhang & Gui-Ping Zhao (HMJAU 48251, holotype, GenBank Acc. nos.: ITS = MW893249, LSU = MW893274, EF-1α = MZ616769).

##### Etymology.

*changchunensis*: referring to Changchun, the location of the holotype.

##### Diagnosis.

*Cordycepschangchunensis* can be easily differentiated from closely-related species *C.militaris* by its unique host, smaller stromata, immersed perithecia and larger part-spores (2.6–6 × 1.0–1.4 μm).

##### Description.

**Sexual Morph. Stromata** 2.4–4.5 cm long, single or multiple, solitary to gregarious, arising from pupa; branched, sometimes single at base, then branched into two forks. **Fertile apical** portion, orange, clavate to globose, sometimes irregular, 2.0–3.5 cm long and 0.4–0.6 cm wide, distinctly distinguishable from the stipe. **Sterile stipe** fleshy, light yellow to orange, cylindrical, 1.3–3.3 cm long and ca. 0.4 cm wide, usually with white mycelium at the base. **Perithecia** immersed at right angles to the surface of the fruiting body, globose to ovoid, 180–600 × 180–520 μm, with a thick wall about 10–15 μm. **Asci** cylindrical, 80–300 × 2.5–5 μm, 8–spored, apex of ascus hemispherical, 3.0–4.0 × 2.0–3.0 μm. **Part-spores** oblong, 2.6–6 × 1.0–1.4 μm, smooth, hyaline in 3% KOH, thin-walled, inamyloid.

**Figure 3.**
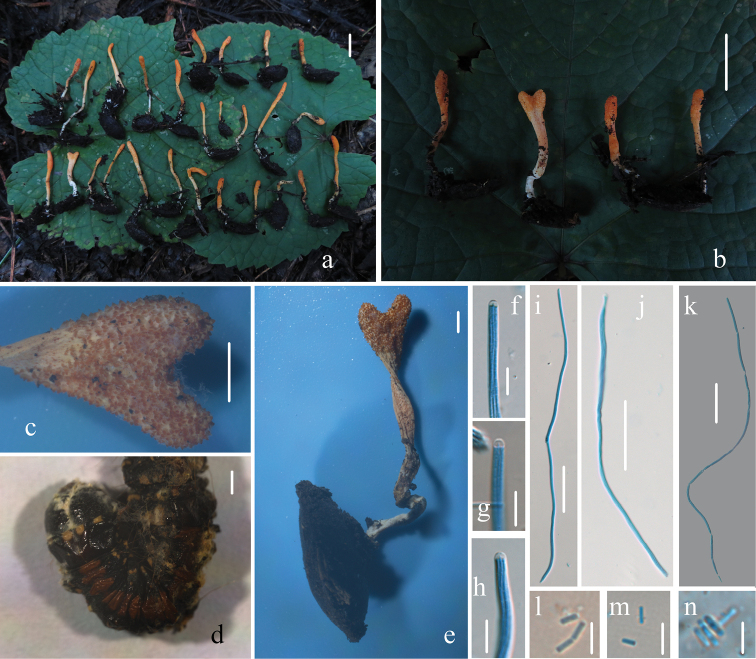
Morphological characters of *Cordycepschangchunensis* (HMJAU 48251, holotype) **a, b, e** stromata and host of *Cordycepschangchunensis***c** surface of fertile apex of ascostroma **d** host of *Cordycepschangchunensis***f–h** apex of ascus **i–k** ascus **l–n** part-spores. Scale bars: 1 cm (**a, b**); 2 mm (**c, e**); 1 mm (**d**); 10 μm (**f–h**); 50 μm (**i–k**); 5 μm (**l–n**).

**Asexual Morph.** Unknown.

##### Host.

Growing on pupae of Lepidoptera.

##### Other specimens examined.

China. Jilin Province: Changchun City, Jingyuetan National Forest Park, 20 August 2015, Bo Zhang (HMJAU 48259, GenBank Acc. nos.: ITS = MW893251, LSU = MW893276, EF-1α = MZ616773); Changchun City, Jingyuetan National Forest Park, 18 August 2018, Bo Zhang (HMJAU 48252, isotype, GenBank Acc. nos.: ITS = MW893250, LSU = MW893275, EF-1α = MZ616775).

##### Distribution.

China (Jilin Province).

##### Note.

*C.changchunensis* is easily confused with *C.militaris* due to highly similar morphology and sharing the same habitat. Morphologically, the stromata of *C.militaris* are larger than *C.changchunensis*, single or gregarious, larger perithecia (500–1089 × 132–264 μm) and smaller part-spores (2–4 × 1 μm) (Li et al. 2015). In the phylogenetic analysis, the three specimens of *C.changchunensis* were placed in separate monophyletic lineages (BPP = 0.91, MLBS = 78%).

**Figure 4.**
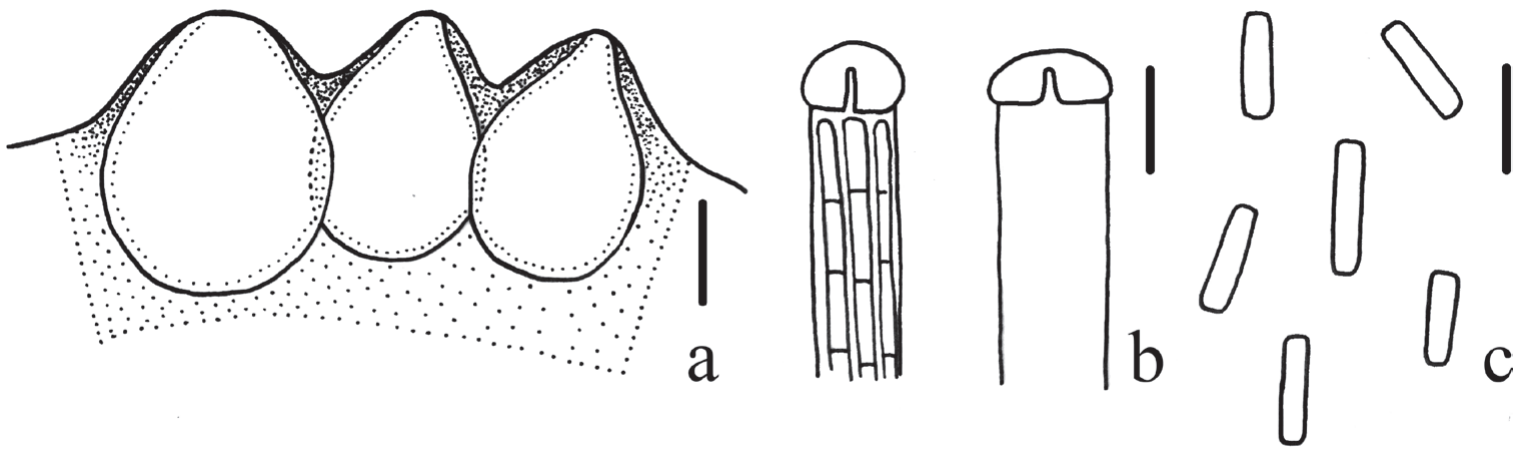
Microscopical characters of *Cordycepschangchunensis* (HMJAU 48251, holotype) **a** perithecia **b** apex of ascus **c** part-spores. Scale bars: 100 μm (**a**); 5 μm (**b, c**).

#### Cordyceps changbaiensis

Taxon classificationFungiHypocrealesCordycipitaceae

J.J. Hu, Bo Zhang & Y. Li sp. nov.

9A01A704-BE44-516F-BC79-06B1EC2B9359

839250

Figs 5 , 6

##### Holotype.

China. Jilin Province, Yanbian Korean Autonomous Prefecture, Antu County, Changbai Mountain, 42.19°N, 128.18°E, 4 September 2019, Jia-Jun Hu & Bo Zhang (HMJAU 48255, holotype, GenBank Acc. nos.: ITS = MW893252, LSU = MW893277, EF-1α = MZ616772).

##### Etymology.

*changbaiensis*: referring to Mt. Changbai, the location of the holotype.

##### Diagnosis.

The species is characterised by orange to white and branched stromata, globose to ovoid perithecia and larger part-spores (3.0–7.0 × 1.0–1.4 μm).

**Figure 5.**
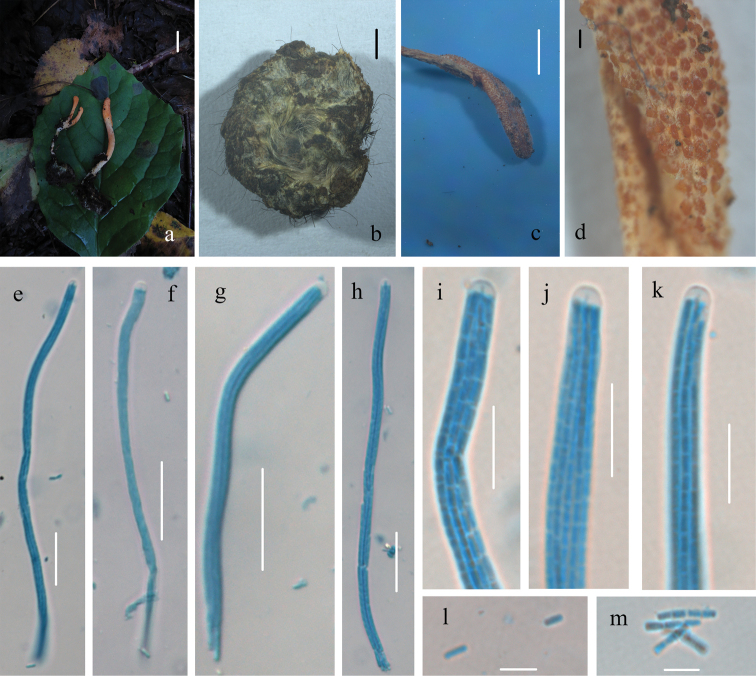
Morphological characters of *Cordycepschangbaiensis* (HMJAU 48255, holotype) **a** stromata and host of *Cordycepschangbaiensis***b** host of *Cordycepschangbaiensis***c, d** surface of fertile apex of ascostroma **e–h** ascus **i–k** apex of ascus **l–m** part-spores. Scale bars: 1 cm (**a**); 5 mm (**b–c**); 200 μm (**d**); 20 μm (**e–h**); 10 μm (**i–k**); 5 μm (**l–m**).

##### Description.

**Sexual Morph. Stromata** 2.4–5.2 cm long, single or multiple, solitary, arising from the head of the host insect covered with white mycelia. **Fertile apical** portion, orange, clavate to cylindrical, 0.6–1.5 cm long and 0.2–0.6 cm wide, obviously distinguishable from the stipe. **Sterile stipe** fleshy, white to light yellow, cylindrical, 1.8–3.7 cm long and 0.2–0.5 cm wide. **Perithecia** immersed to the surface of the fruiting body, globose to ovoid, 120–230 × 90–170 μm, with a thick wall about 15 μm. **Asci** cylindrical, 225–625 × 4–5 μm, 8–spored, apex of ascus hemispherical, 3.0–4.0 × 2.2–3.2 μm. **Part-spores** oblong, 3.0–7.0 × 1.0–1.4 μm, smooth, hyaline in 3% KOH, thin-walled, inamyloid.

**Figure 6.**
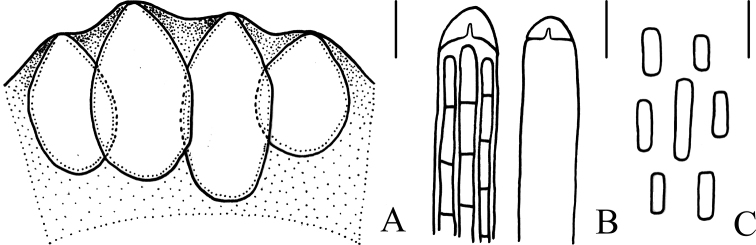
Microscopical characters of *Cordycepschangbaiensis* (HMJAU 48255, holotype) **a** perithecia **b** apex of ascus **c** part-spores. Scale bars: 100 μm (**a**); 5 μm (**b, c**).

**Asexual Morph.** Unknown.

##### Host.

Growing on larvae of Lepidoptera.

##### Distribution.

China (Jilin Province).

##### Other specimen examined.

China. Jilin Province: Baishan City, Fusong County, Quanyang Town, 42.30°N, 127.29°E, 22 August 2021, Jia-Jun Hu, Bo Zhang & Gui-Ping Zhao (HMJAU 482260, isotype, GenBank Acc. nos.: ITS = MW893270, LSU = MW893272, EF-1α = MZ616774)

##### Note.

*C.changbaiensis* has orange to white and branched stromata. Morphologically, *C.roseostromata* Kobayasi & Shimizu is similar to *C.changbaiensis* due to the single or branched stromata. *C.kyushuensis* A. Kawam. is also close to *C.changbaiensis* because of the host and the stromata being similar in colour. However, both *C.roseostromata* and *C.kyushuensis* have a larger perithecia and smaller part-spores. Furthermore, the stromata of *C.kyushuensis* is gregarious or fascicled and grows from the head or abdomen of the host (Li et al. 2015); *C.roseostromata* has pyriform perithecia and host on larva of Coleoptera (Kobayasi 1983). In the phylogenetic analysis, *C.changbaiensis* was placed in separate monophyletic lineages (BPP = 0.95, MLBS = 97%) and formed a sister relationship with *C.rosea*.

#### Cordyceps jinyuetanensis

Taxon classificationFungiHypocrealesCordycipitaceae

J.J. Hu, Bo Zhang & Y. Li sp. nov.

4DF39943-86BC-5BC9-8F39-D40810050897

839251

Figs 7 , 8

##### Holotype.

China. Jilin Province: Changchun City, Jingyuetan National Forest Park, 43.80°N, 125.50°E, 27 August 2018, Jia-Jun Hu, Bo Zhang & Gui-Ping Zhao (HMJAU 48253, holotype, GenBank Acc. nos.: ITS = MW893253, LSU = MW893278, EF-1α = MZ616770).

##### Etymology.

*jinyuetanensis*: referring to Jingyuetan National Forest Park, the location of the holotype.

##### Diagnosis.

*C.jingyuetanensis* is different from other species by growing on pupae, orange to light red stromata, immersed and almond-shaped to ovoid perithecia.

**Figure 7.**
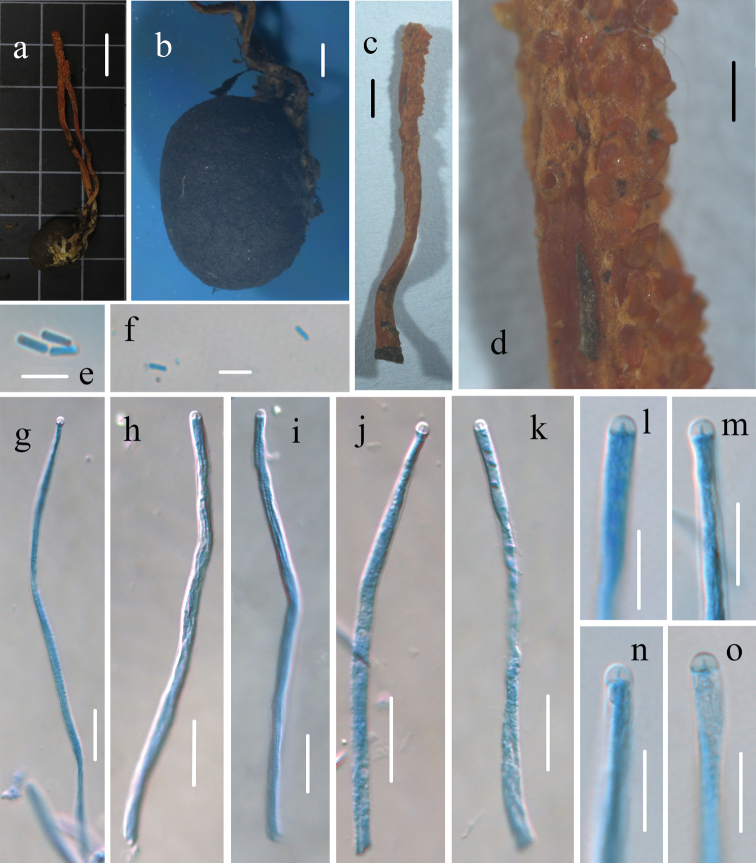
Morphological characters of *Cordycepsjingyuetanensis* (HMJAU 48253, holotype) **a** stromata and host of *Cordycepsjingyuetanensis***b** host of *Cordycepsjingyuetanensis***c, d** surface of fertile apex of ascostroma **e, f** part-spores **g–k** ascus **l–o** apex of ascus. Scale bars: 1 cm (**a**); 2 mm (**b, c**); 500 μm (**d**); 5 μm (**e, f**); 20 μm (**g–k**); 10 μm (**i–o**).

##### Description.

**Sexual Morph. Stromata** 4–4.5 cm long, multiple, solitary, arising from pupae of Lepidoptera. **Fertile apical** portion, orange to light red, clavate, 0.8–1.3 cm long and 0.1–0.2 cm wide, obviously distinguishable from the stipe. **Sterile stipe** fleshy, light yellow to orange, cylindrical, 2.7–3.7 cm long and 0.1–0.2 cm wide, usually with white mycelium at the base. **Perithecia** immersed to the surface of the fruiting body, almond-shaped to ovoid, 220–340 × 110–220 μm, with a thick wall about 15–20 μm. **Asci** cylindrical, 225–475 × 3–5 μm, 8-spored, apex of ascus hemispherical to irregular, 3.0–4.0 × 1.4–2.8 μm. **Part-spores** oblong, 2.8–5.0 × 1.0–1.4 μm, smooth, hyaline in 3% KOH, thin-walled, inamyloid.

**Figure 8.**
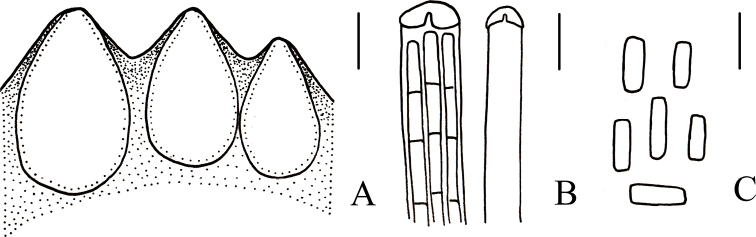
Microscopical characters of *Cordycepsjingyuetanensis* (HMJAU 48253, holotype) **a** perithecia **b** apex of ascus **c** part-spores. Scale bars: 100 μm (**a**); 5 μm (**b, c**).

**Asexual Morph.** Unknown.

##### Host.

Growing on pupae of Lepidoptera.

##### Distribution.

China (Jilin Province).

##### Other specimen examined.

China. Jilin Province: Baishan City, Fusong County, Quanyang Town, 42.30°N, 127.29°E, 22 August 2021, Jia-Jun Hu, Bo Zhang & Gui-Ping Zhao (HMJAU 482261, isotype, GenBank Acc. nos.: ITS = MW893271, LSU = MW893273)

##### Note.

A review of literature revealed that there are about 20 species of Cordycipitiod fungi growing on pupae, like the unusual medicinal fungi *O.sinensis* (Berk.) G.H. Sung, J.M. Sung, Hywel-Jones & Spatafora, *C.militaris*, *I.cicadae* Miq. and also like the two new species, *C.ningxiaensis* T. Bau & J.Q. Yan and *C.qingchengensis* L.S. Zha & T.C. Wen, reported from China in 2015 and 2019. Nevertheless, *C.jingyuetanensis* is different from these Cordycipitiod species; *C.ningxiaensis* grows on the pupae of Diptera, *I.cicadae* grows on the pupae of Hemiptera and the stromata of *C.qingchengensis* are yellow, single or branched on the top. *C.hepialidicola* Kobayasi & Shimizu from Japan is similar to *C.jingyuetanensis* in its phylogenetic relationship, but there are distinct morphological differences. Morphologically, the stromata of *C.hepialidicola* are multiple, branched on the top sometimes, grow from the head of larva of Hepialida or Lepidoptera, have larger perithecia (300–350 × 500 μm) and smaller part-spores (3–4 × 1 μm) (Kobayasi 1983). In the phylogenetic analysis, *C.changbaiensis* was placed in separate monophyletic lineages (BPP = 0.92, MLBS = 79%).

#### Cordyceps militaris

Taxon classificationFungiHypocrealesCordycipitaceae

(L.) Fr., Observ. mycol. (Havniae) 2: 317 (cancellans) (1818)

537EF178-CAB4-5CF7-A165-0471C3805266

Fig. 9

##### Specimens examined.

China. Yunnan Province: Qujin City, Huize County, 26.24°N, 103.25°E, 30 July 2019, Jia-Jun Hu, Bo Zhang & Di-Zhe Guo (HMJAU 48256, GenBank Acc. nos.: ITS = MW888227, LSU = MW893279); Jilin Province: Changchun City, Jingyuetan National Forest Park, 43.80°N, 125.50°E, 25 August 2018, Jia-Jun Hu & Yong-Lan Tuo (HMJAU 48257); Changchun City, Jingyuetan National Forest Park, 43.80°N, 125.50°E, 25 August 2018, Jia-Jun Hu, Bo Zhang & Gui-Ping Zhao (HMJAU 48258); Tonghua City, Ji’an County, Wunvfeng National Forest Park, 41.28°N, 126.14°E, 25 August 2019, Yong-Lan Tuo (HMJAU 48262); Heilongjiang Province: Daxing’an Mountains, Shuanghe National Nature Reserve, 52.44°N, 125.40°E, 23 June 2019, Di-Zhe Guo (HMJAU 48263).

**Figure 9.**
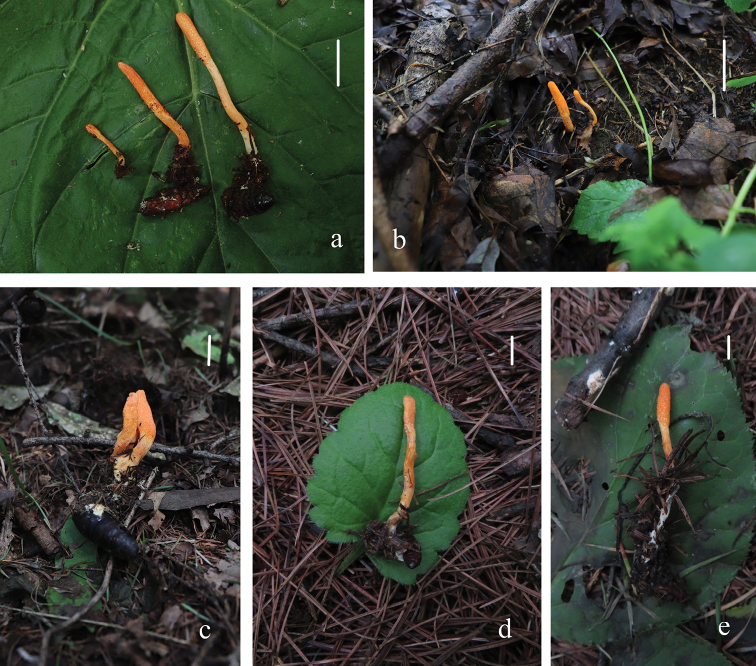
Macrocharacter of *Cordycepsmilitaris***a–e** stromata and host of *Cordycepsmilitaris* (**a** collected from Daxing’an Mountains, Heilongjiang Province **b** collected from Ji’an County, Tonghua City, Jilin Province **c, e** collected from Changchun City, Jilin Province **d** collected from Qujin City, Huize County, Yunnan Province). Scale bars: 1 cm (**a–e**).

##### Note.

*C.militaris* is a widely distributed species and also a well-known medicinal fungus in China. At this time, we collected samples from many different places. The morphological evidence shows no apparent differences between each other. However, the habitat is markedly different.

**Table 2.** Morphological comparisons of sexual states of *Cordycepschangchunensis*, *Cordycepschangbaiensis* and *Cordycepsjingyuetanensis*.

Species	Host	Stromata	Fertile part	Perithecia	Asci	Ascospores	Reference
* Beauveria bassiana *	Larvae of Lepidoptera	Single or several, unbranched, slender and cylindrical, brownish- yellow to yellowish	18.7–33. 3 × 2.8–8.0 mm	Elliptical, 610–720 × 230–320 µm, immersed to surface	Cylindrical, 230–590 × 3.5–4.0 µm with ascus cap 3.6–4.0 µm in diameter	Filamentous, 300–570 × 1.0 µm, not broken into part-spores	(Li et al. 2001)
* Blackwellomyces pseudomilitaris *	Larvae of Lepidoptera	Single or cluster, simple or branched, cylindrical, white to white-orange	15–30 × 0.9–3 mm	Elongate-ellipsoid or elongate-ovoid, 290–570 × 120–245 µm, superficial	Filiform, 290–410 × 5–6 µm	Filiform, 280–390 × 1 µm, not broken into part-spores	(Hywel-Jones 1994)
* Cordyceps bifusispora *	Larvae of Lepidoptera	Simple, cylindrical clavate, whitish	6 × 1.3 mm	Pyriform, with protruding apices, yellowish, 300 × 150–170 µm, immersed	Cylindrical, 200–220 × 3–4.5 µm	Bifusiform, 145–220 µm in length, central part filiform about 0.4 µm wide, terminal parts narrowly fusiform, about 30 × 1.6 µm and 3 septate	(Eriksson 1982)
* C. kyushuensis *	Larvae of Lepidoptera	Cluster, cylindrical, Light yellow to orange red	20–30 × 5–8 mm	Elliptical, 300–500 × 200–300 µm, half-buried	Cylindrical, 3–4.5 µm wide	Short cylindrical, part-spores 5–7 × 0.7–1 µm	(Guo and Li 2000; Li et al. 2015)
* C. militaris *	Lepidopteran pupae	Single or several, clavate, orange	10–20 × 3–5 mm	Conical, half-buried	Clavate, 300–400 × 4–5 µm	Filiform, part spores 2–3 × 1 µm	(Li et al. 2015)
* C. ningxiaensis *	Fly pupae (Diptera)	One to two in a group, clavate, orange	1.2–3 × 1.2–2.8 mm	Ellipsoid to ovoid, 288–400 × 103–240 μm, with a wall about 10 μm thick, loosely embedded at right angles to the surface	Cylindrical, 168–205 × (3.7–)4.1–5.5(–6.6) μm, with oblate spheroid or hemispherical refractive cap 3.4–3.8 × 2.9–3.4 μm at apex	Filiform, irregularly multiseptate, part-spores 3.6–7.8 × 1.0–1.4 μm	(Yan and Bau 2015)
* C. polyarthra *	Larvae of Lepidoptera	Cespitose, narrowly clavate, light yellow to reddish-brown		Ovoid, 250–450 × 125–250 μm, brown, with a definite wall 25 μm thick, embedded at right angles to the surface	Cylindrical, 150–260 × 3–4 μm, with a 1.5–2 μm thick cap	Filiform, part-spores 4–6 × 0.75–1 μm	(Mains 1958)
* C. pruinosa *	Larvae of Lepidoptera	Solitary or several, clavate, orange to red	2–8 × 1–3 mm	Ovoid to fusiform, 360–400 × 130–200 μm, crowded, red, ordinal in orientation, immersed	Cylindrical, 100–200 × 2.5–4 μm	Filiform, part-spores 4–6 × 1 μm	(Li et al. 2015)
* C. qingchengensis *	Lepidopteran pupae	Branched, yellow	7–9 × 2.0–2.5 mm	Ovoid but apex sharply pointed, 335–490 × 145–240 μm, partially immersed at right angle to the surface	Cylindrical, 180–200 × 2.4–4.0 μm wide, caps hemispherical, 1.8–2.2 × 2.5–3.2 μm	Filiform, 180–220 × 0.45–0.65 μm, not at all bifusiform and not broken into part-spores	(Zha et al. 2019)
* C. roseostromata *	Larva, not specified	Single or branched	1.2–5 × 1.5–2.2 mm	Pyriform, 280–300 × 140–160 μm, Superficial	3–3.5 × 2.5–3 μm	4–5 × 1 μm	(Kobayasi 1983)
* C. changchunensis *	Lepidopteran pupae	Single or multiple, clavate, orange	2.0–3.5 × 0.4–0.6 mm	Globose to ovoid, 180–600 × 180–520 μm, with a thick wall about 10–15 μm, partially immersed at right angles to the surface	Cylindrical, 80–300 × 2.5–5 μm, caps hemispheric, 3.0–4.0 × 2.0–3.0 μm at apex	Oblong, 2.6–6 × 1.0–1.4 μm	This study
* C. changbaiensis *	Larvae of Lepidoptera	Single or multiple, clavate, white to orange	0.6–1.5 × 0.2–0.6 mm	Globose to ovoid, 120–230 × 90–170 μm, with a thick wall about 15 μm, immersed to surface	Cylindrical, 225–625 × 4–5 μm, caps hemispherical, 3.0–4.0 × 2.2–3.2 μm at apex	Oblong, 3.0–7.0 × 1.0–1.4 μm	This study
* C. jingyuetanensis *	Lepidopteran pupae	Single or multiple, clavate, orange to light red	0.8–1.3 × 0.1–0.2 mm	Almond-shaped to ovoid, 220–340 × 110–220 μm, with a thick wall about 15–20 μm, immersed to surface	Cylindrical, 225–475 × 3–5 μm, caps hemispherical to irregular, 3.0–4.0 × 1.4–2.8 μm at apex	Oblong, 2.8–5.0 × 1.0–1.4 μm	This study

### Key to reported species in this study

**Table:** 

1	Stromata arise from pupae	**2**
–	Stromata arise from larvae	*** Cordyceps changbaiensis ***
2	Stromata branched into two forks sometimes	*** Cordyceps changchunensis ***
–	Stromata not branched	**3**
3	Part-spores over 3 μm	*** Cordyceps jingyuetanensis ***
–	Part-spores less than 3 μm	*** Cordyceps militaris ***

## Discussion

In this study, three new species, collected from northeast China in the *Cordycepsmilitaris* group, are described. In previous work, about 38 species were recognised as belonging to the *C.militaris* group (Yan and Bau 2015). ML and BI analysis recognised four well-supported clades, one is Cordycipitaceae, the others are Clavicipitaceae and Ophiocordycipitaceae (Fig. 2). Moreover, the Cordycipitaceae branch is mainly divided into three clades, the *Akanthomyces* clade near the *Cordyceps* clade, implies a closer biological relationship.

The previous studies have revealed that the genus *Cordyceps* was not monophylic (Artjariyasripong et al. 2001), the species of *Isaria* was nested within *Cordyceps* (Kepler et al. 2017) and our phylogenetic analysis also shows a similar result. *Cordyceps* clade consisted of three major subclades designated as clade 1, clade 2 and clade 3 (Fig. 2). Nearly all the subclades in *Cordyceps* clade were strongly supported.

Clade 1, including nine *Cordyceps* spp. and two *Isaria* spp. *I.cicadae*, based on Chinese sequences, gathers into one branch with *Cordyceps* species. What is known as *I.cicadae* in China, named on a Brazilian specimen, is of confused classification status, due to the teleomorph having remained undiscovered. In China, *C.cicadae* Massee has been regarded as a teleomorph of *I.cicadae* as well as a teleomorph of *O.sobolifera* (Hill ex Watson) G.H. Sung, J.M. Sung, Hywel-Jones & Spatafora and referred to as *C.sobolifera* (Hill ex Watson) Berk. & Broome. Until recently, the teleomorph was discovered in Mt. Jinggang, Jiangxi Province, China and both teleomorph and anamorph existed on some specimens, with the morphology of the anamorph consistent with those, “*I.cicadae*”, harvested throughout southern China, significantly different from the type specimen of *I.cicadae*. For this reason, it was published as a new species named *C.chanhua* Z.Z. Li, F.G. Luan, Hywel-Jones, C.R. Li & S.L. Zhang (Zhi et al. 2021). Furthermore, *I.japonica* Yasuda reported from Japan, exhibits exceptionally high affinity with the genus *Cordyceps*. The teleomorph, however, still remains a mystery and a more intensive study is needed. Clade 2 consists of *C.scarabaeicola* Kobayasi, *C.bassiana* Z.Z. Li, C.R. Li, B. Huang & M.Z. Fan and *C.brongniartii* Shimazu. Yellow stromata seem to be a synapomorphic character of clade 2. Clade 3 included 15 *Cordyceps* spp. However, clade 3 did not form a monophyletic group. *C.ninchukispora* (C.H. Su & H.H. Wang) G.H. Sung, J.M. Sung, Hywel-Jones & Spatafora, *C.chiangdaoensis* Tasan., Thanakitp., Khons. & Luangsa-ard, *C.pruinosa* Petch and *C.morakotii* Tasan., Thanakitp. & Luangsa-ard gather into one branch. *Cordyceps* spp. of clade 3A all arise from pupae. Clade 3B includes 11 *Cordyceps* spp., seven known *Cordyceps* spp., one unidentified *Cordyceps* sp. and our three new species. Being visually similar to *Cordycepsmilitaris* seems to be a synapomorphic character of clade 3B.

About 60% of *Cordyceps* sensu lato species are recorded on two insect orders–Coleoptera and Lepidoptera (Shrestha et al. 2016). Host preferences have been variously implemented in taxonomic work, so this is also in *Cordyceps*. Host associations, when superimposed on phylogeny, suggested that some groups of taxa have conserved the endoparasite-host interactions to some extent; however, several host shifts have occurred during the evolution of *Cordyceps* (Stensrud et al. 2005). In *Cordyceps* species, hosts were considered as having low significance as a phylogenetic character, but are the most crucial feature in morphological aspects (Torres et al. 2005).

## Supplementary Material

XML Treatment for Cordyceps changchunensis

XML Treatment for Cordyceps changbaiensis

XML Treatment for Cordyceps jinyuetanensis

XML Treatment for Cordyceps militaris
